# Efficacy of PermaNet^® ^2.0 and PermaNet^® ^3.0 against insecticide-resistant *Anopheles gambiae *in experimental huts in Côte d'Ivoire

**DOI:** 10.1186/1475-2875-10-172

**Published:** 2011-06-23

**Authors:** Benjamin G Koudou, Alphonsine A Koffi, David Malone, Janet Hemingway

**Affiliations:** 1 Département Environnement et Santé, Centre Suisse de Recherches Scientifiques, 01 BP 1303 Abidjan 01, Côte d'Ivoire; 2 UFR Sciences de la Nature, Université d'Abobo-Adjame, 02 BP 801 Abidjan 02, Côte d'Ivoire; 3 Institut Pierre Richet, 01 BP 1500 Bouaké 01, Côte d'Ivoire; 4 Liverpool School of Tropical Medicine, Pembroke Place, Liverpool, L3 5QA, UK

## Abstract

**Background:**

Pyrethroid resistance in vectors could limit the efficacy of long-lasting insecticidal nets (LLINs) because all LLINs are currently treated with pyrethroids. The goal of this study was to evaluate the efficacy and wash resistance of PermaNet^® ^3.0 compared to PermaNet^® ^2.0 in an area of high pyrethroid in Côte d'Ivoire. PermaNet^® ^3.0 is impregnated with deltamethrin at 85 mg/m^2 ^on the sides of the net and with deltamethrin and piperonyl butoxide on the roof. PermaNet^® ^2.0 is impregnated with deltamethrin at 55 mg/m^2 ^across the entire net.

**Methods:**

The study was conducted in the station of Yaokoffikro, in central Côte d'Ivoire. The efficacy of intact unwashed and washed LLINs was compared over a 12-week period with a conventionally-treated net (CTN) washed to just before exhaustion. WHO cone bioassays were performed on sub-sections of the nets, using wild-resistant *An. gambiae *and Kisumu strains. Mosquitoes were collected five days per week and were identified to genus and species level and classified as dead or alive, then unfed or blood-fed.

**Results:**

Mortality rates of over 80% from cone bioassays with wild-caught pyrethroid-resistant *An. gambiae *s.s were recorded only with unwashed PermaNet^® ^3.0. Over 12 weeks, a total of 7,291 mosquitoes were collected. There were significantly more *An. gambiae *s.s. and *Culex *spp. caught in control huts than with other treatments (P < 0.001). The proportion of mosquitoes exiting the huts was significantly lower with the control than for the treatment arms (P < 0.001). Mortality rates with resistant *An. gambiae *s.s and *Culex *spp, were lower for the control than for other treatments (P < 0.001), which did not differ (P > 0.05) except for unwashed PermaNet^® ^3.0 (P < 0.001), which gave significantly higher mortality (P < 0.001).

**Conclusions:**

This study showed that unwashed PermaNet^® ^3.0 caused significantly higher mortality against pyrethroid resistant *An. gambiae s.s *and *Culex *spp than PermaNet^® ^2.0 and the CTN. The increased efficacy with unwashed PermaNet^® ^3.0 over PermaNet^® ^2.0 and the CTN was also demonstrated by higher KD and mortality rates (KD > 95% and mortality rate > 80%) in cone bioassays performed with wild pyrethroid-resistant *An. gambiae s.s *from Yaokoffikro.

## Background

Despite considerable efforts to control malaria, the disease remains one of the most pressing public health issues across sub-Saharan Africa. In 2006, there were about 250 million malaria cases [1]. In 2008, there were an estimated 880,000 deaths from malaria [2,3]. Insecticide-treated nets (ITNs) significantly reduce malaria-related morbidity and mortality [4], and are increasingly utilized in sub-Saharan Africa. In Côte d'Ivoire, ITN coverage is low (<10%), with the majority of nets being considered as 'untreated' as there is evidence that the insecticide used for re-treatment is often not applied within the recommended six months time frame [4]. In resource-constrained settings, such as Côte d'Ivoire, the relatively short period those insecticides remain effective on a conventionally treated net (CTN) is an important obstacle to sustaining high coverage rates of ITNs.

Long-lasting insecticidal nets (LLINs) were developed as a more sustainable solution to the problems encountered with CTNs and are currently expected to retain biological activity for at least 20 standard World Health Organization (WHO) washes under laboratory conditions and three years of use under field conditions. There are two types of technology for manufacturing LLINs: incorporating the insecticide into polyethylene or polypropylene yarn or coating the insecticide onto polyester. PermaNet^® ^2.0 is a WHO recommended polyester LLIN coated with the pyrethroid deltamethrin to a target dose of 55 mg/m^2 ^(±25%). This net has been tested in multiple laboratories and field sites. PermaNet^® ^3.0 was designed to give increased bio-efficacy against pyrethroid-resistant malaria vectors; it is made from two types of fabric; deltamethrin coated on the polyester sides of the net and a synergist piperonyl butoxide (PBO) incorporated with deltamethrin in the polyethylene roof. The target dose of deltamethrin in the sides of PermaNet^® ^3.0 is 85 mg/m^2 ^(±25%).

Pyrethroid resistance can be caused by high frequency of kdr and/or enhanced metabolic mechanisms in *Anopheles gambiae *s.s. These types of mechanisms are widespread in West Africa and in some cases have been shown to reduce the efficacy of treated nets [5,6]. It is therefore essential that new LLINs that are efficient against insecticide-resistant malaria vectors are developed. The goal of this study was to evaluate the efficacy of PermaNet^® ^3.0 against insecticide-resistant *An. gambiae *s.s, *Culex *sp and susceptible *Mansonia *sp in experimental huts in Côte d'Ivoire using washed and unwashed nets. *Mansoni*a was included during this study as it presents a significant biting nuisance in Côte d'Ivoire [7,8].

## Methods

### Study area and experimental huts

The experimental hut station was located at Yaokoffikro, near Bouaké in central Côte d'Ivoire, where 18 experimental huts are available for the assessment of new insecticide formulations. Six experimental huts, built with bricks coated in cement, were refurbished before starting the trial. Mosquito vector abundance at the field site is dominated by *An. gambiae *s.s and *Culex *sp; the majority of the *An. gambiae *s.s. population is represented by the S form (90%) and M form (10%) at low frequency, with high kdr frequencies (94%) combined with a lower frequency of P450-based metabolic resistance [9,10].

The huts were situated near rice and vegetable fields in two rows with a five-meter gap between huts. The style of the hut was typical of the region, made from concrete bricks, with a corrugated iron roof, a ceiling of thick polyethylene sheeting, and a concrete base surrounded by a water-filled channel to prevent entry of ants [11]. Mosquito access was via 4 window slits constructed from pieces of plywood, fixed at an angle to create a tunnel with a 1 cm wide gap. Mosquitoes had to fly upward to enter through the gap and downwards to exit; this limited exodus though the aperture enabling the majority of entering mosquitoes to be retained. A veranda trap made of polyethylene sheeting and screening mesh measuring 2 m long, 1.5 m wide, and 1.5 m high, projected from the back wall of each hut. Movement of mosquitoes between hut and veranda was unimpeded. All huts were thoroughly cleaned before the trial. Sheets were laid over the floor each night to facilitate the collection of mosquitoes from the floor each morning.

### Net treatment and washing procedures

The conventionally-treated nets (CTNs) were dipped and washed alongside the LLINs at the Liverpool School of Tropical Medicine (LSTM) according to standard WHO procedures described below. The standardized WHO recommended washing method used is not meant to simulate washing procedures in real-life, but allows comparisons with other studies using this format of washing.

#### Conventionally-treated nets washed to just before exhaustion

Untreated polyester nets were treated with K-O TAB^® ^(deltamethrin 1% corresponding to a dose of 25 mg/m^2^) following the manufacturer's instructions (Bayer Environmental Sciences). The point of exhaustion was determined by treating one net and performing WHO cone bioassays after each wash, until reaching the exhaustion point, defined as the last wash for which the net still causes ≥80% mortality or ≥ 95% knockdown (KD). After the sixth wash, the mortality rate against the standard susceptible *An. gambiae s.s*. Kisumu strain was under 80% and the KD rate was below 95%. Consequently, five nets were treated with K-O TAB^® ^and washed five times. After the fifth wash the mortality and the KD rates of the dipped nets were ≥80% and ≥ 95%, respectively, compared to those recorded with the unwashed CTN, hence the targeted dose of the dipped net was assumed to be approximately 25 mg/m^2^.

#### Washing procedure

Before each washing of each net (LLINs and CTNs), 20 g of "Savon de Marseille" (Unilever) was added to 10 l of de-chlorinated water and fully dissolved for 30 minutes. Each net was washed, immersed in the soap solution and manually agitated by hand (with protection by gloves) for 10 minutes at approximately 20 rotations per minute. Nets were then rinsed twice in fresh tap water and dried horizontally in shade. The nets were stored at ambient temperature between washes; CTNs were washed five times and PermaNet^® ^2.0 and PermaNet^® ^3.0 were washed 20 times with a one day interval between washes.

### Bioassays

Insecticide-susceptible *An. gambiae *s.s. used in bioassays were from a Kisumu strain established at Institut Pierre Richet. Insecticide-resistant *An. gambiae *s.s adults were obtained from larval collections from natural breeding sites in Yaokoffikro and then transported to the laboratory for rearing to adults. All larvae were provided a diet of Tetra Mikromin fish food until adult emergence. All mosquito rearing and bioassays were performed under ambient environmental conditions.

Cone bioassays were performed at the beginning and at the end of the trial. Susceptible and resistant *An. gambiae *s.s strains were tested separately. Batches of 5-6 unfed, 2-5 day old female *An. gambiae *s.s. including the standard susceptible Kisumu strain and a wild insecticide-resistant adult mosquitoes were placed inside the WHO cone and exposed simultaneously and in a similar manner to different parts of the unwashed and washed nets (the roof and the sides) for three minutes before being transferred from cones to holding containers. The number of mosquitoes knocked down was then recorded 60 minutes after exposure and the final mortality was recorded 24 hours post-exposure. Survivors were maintained on 10% honey solution. An untreated net was used as a negative control.

WHO susceptibility tests were performed on 3-5 day old unfed wild-caught pyrethroid-resistant females reared from larval collections, using standard WHO test kits and protocols for adult mosquitoes [12]. In brief, papers impregnated with 0.05% deltamethrin, 0.75% permethrin and 4% DDT were sourced from WHO. Batches of 20--25 females were exposed to impregnated papers in WHO test tubes for 1 h with at least four replicates per bioassay and concurrent negative controls with corresponding insecticide-free papers. Knockdown (KD) was recorded after 60 min and mosquitoes were transferred to holding containers with access to a 10% honey solution. Mortality was recorded after 24 h.

### Volunteer sleepers, rotation and mosquito collection

Preliminary results showed no significant difference in attractiveness of different huts as there was no statistical difference between the mean number of mosquitoes collected in huts after sleepers rotation (P = 0.05). The following 6 treatment arms were tested:

• Untreated net (control)

• Unwashed PermaNet^® ^2.0

• Unwashed PermaNet^® ^3.0

• PermaNet^® ^2.0 washed 20x

• PermaNet^® ^3.0 washed 20x

• Conventionally-treated net (with deltamethrin) washed 5x.

Five nets were used for each treatment arm with each net being tested on one night during each week. Holes were not cut in the nets for ethical reasons as requested by the National Malaria Control Programme. Treatment arms were rotated among the huts each week, according to a pre-established Latin square design. Five adult male volunteers slept in the huts from 20:00 to 05:00 h each night and were rotated randomly among huts each night of the study. At the end of each week the huts were cleaned and aired to avoid potential contamination.

The trial lasted 12 weeks (from April to July 2009) as the number of mosquitoes collected during the first 6 weeks of the trial was low. Each morning, mosquitoes were collected from the floor, walls and roof of the huts as well as from the exit traps. Collected mosquitoes were transferred to the nearby laboratory and identified to the genus and species level using a determination key [13], and classified as dead/blood fed, alive/blood fed, dead/unfed, and alive/unfed. Surviving mosquitoes were provided with honey solution and held for 24 h after which delayed mortality was assessed.

The effect of each treatment was expressed relative to the control (untreated net) by assessing the deterrent effect (proportion entering), which shows the dissuasive effect of the treatment arms, the induced exophily (proportion of mosquitoes that exit early through the exit traps, treatment-induced exiting) and mortality rates. The blood feeding rate could not be measured as a standard parameter as the number of blood fed *An. gambiae *s.s collected was too low and holes were not included in the nets as per standard WHOPES protocol [12]. All parameters were reported separately for each treatment.

### Ethical considerations

Ethical approval was obtained from the Ministry of Health, Côte d'Ivoire, through the National Malaria Control Programme. Written informed consent was obtained from each volunteer sleeper, who was offered anti-malarial chemotherapy (artesunate plus amodiaquine) free of charge, and their vaccination status against yellow fever was checked before enrolment. Volunteer sleepers were medically supervised throughout the study by qualified medical personnel.

### Statistical analysis

Data collected every week were double-entered and cross-checked in Windows Excel 2008. Statistical analysis was carried out with STATA version 9.04 (Stata Corporation; College Station, TX, USA) using a significance level of 5%. Data were collected for 4 mosquito species (or groups of species) caught in huts with six types of net (treatments) over a series of 5 nights before changing the treatment allocation the next week. The data variables measured were: number of mosquitoes caught in the room and on the veranda, number dead either immediately after exposure to the net or 24 hrs post exposure and the number of blood fed mosquitoes.

Three main variables were analysed for each mosquito species:

- total number of each mosquito species caught: calculated as (number in room) + (number on veranda)

- proportion of mosquitoes exiting the room: calculated as (number caught on veranda)/(total number)

- mortality: calculated as (total number dead = immediate + delayed)/(total number)

The blood feeding rate was also calculated (number blood fed/total number), but this has not been included as a standardized parameter because holes were not in the nets for ethical reasons, as explained previously. Mosquito numbers were analysed using Poisson GLM (generalized linear model) with a log link function. This model accounts for the variance heterogeneity seen with count data, especially for very low mosquito counts, and fits a model that is linear on the logarithmic scale (exponential in terms of counts). The ratio of all treatments to control is then compared using this model.

The remaining variables were proportion data, and were analysed using a Binomial GLM with logit link function. This model accounts for variance heterogeneity that depends both on the proportion and total number of mosquitoes of each species observed. It fits a model that is linear on the logistic scale (an S-shaped curve on the proportion scale). It is not straightforward to derive ratios between treatments using this model, but it is possible to derive the odds ratio for any test treatment against the control. An odds ratio of 0.5 or less indicates no difference between the treatments; and between treatment and control; an odds ratio > 0.5 indicates a higher proportion for the test treatment compared to the control.

Two sets of analyses were conducted; the first analysis used the daily data to check whether there was any evidence of significant differences between sleepers. This analysis used the model terms: 'Hut' + 'Week' + 'Sleeper' + 'Treatment', where 'Hut' is a factor labelling huts 1-6, 'Week' is a factor labelling weeks 1-12, 'Sleeper' is a factor labelling Sleepers 1-6 and 'Treatment' is a factor labelling treatments 1-6. Fitting this model in this order means allows means for each hut and week to be removed before looking for differences between Sleepers, and treatment differences are assessed only after removing these other effects.

The first analyses showed evidence of sleeper effects only for the blood feeding rate, where numbers were so low as to be unreliable. Hence this parameter could not be used as an outcome measure. The analysis of weekly totals was almost identical to the analysis of daily totals. The statistical analysis on the number of blood fed mosquitoes was then done by using the total blood feds of each species with each treatment, which were analysed separately.

A second set of analyses on weekly totals was therefore carried out using the model terms:

The significance of the treatments was assessed using the F-test from analysis of deviance (analogous to ANOVA for continuous data). Predictions for each treatment were obtained with a 95% confidence interval, and the ratio (or odds ratio) with respect to the control treatment was obtained, again with a 95% confidence interval. Finally, the deterrent effect (proportion entering), the induced exophily and mortality rates between treatment arms were compared.

## Results

### Mosquito abundance

From April to July, a total of 7,291 mosquitoes were collected during 360 man-nights by human volunteer sleepers in 6 huts in the experimental site of Yaokoffikro. Of the mosquitoes caught, 47.1% were *Mansonia *spp, 31.4% were *An. gambiae *s.s, 18.0% were *Culex *spp, 0.6% were *Anopheles funestus *and 2.3% were *Anopheles *species other than *An. gambiae *s.s. or *An. funestus*. The remaining 0.6% of the mosquitoes caught belonged to the genus *Aedes*. There were significantly more *An. gambiae *s.s and *Culex *spp caught in control huts with the untreated nets than with other treatments (P < 0.001) (Additional files 1 and 2, Tables S1 and S2). There was no significant difference in numbers caught for *Mansonia *spp. (P > 0.05). Excluding the untreated net, the numbers of *An. gambiae *s.s *and Culex *spp. collected with each treatment did not differ significantly (P > 0.05).

### Deterrent effect

In comparison to the controls, the entry rate of *An. gambiae *s.s in huts with PermaNet^® ^and the CTNs was reduced by between 60 and 65% (Additional files 1, 2 and 3), with no statistical difference between treatments.

### Induced exophily rate

For *An. gambiae *s.s., *Culex *spp and *Mansonia *spp, significantly more mosquitoes exited in the veranda traps of the huts with PermaNet^® ^and the CTN than in the hut with the untreated control (P < 0.001), but there was no significant difference in the induced exophily rates of *An. gambiae *s.s., *Culex *spp and *Mansonia *spp between the different treatment arms (P > 0.05).

### Mortality

Low mortality rates were recorded in the control hut for *An. gambiae *s.s (6.8%), for the other *Anopheles *species (7.5%) and also for *Culex *spp (6.6%). However, the mortality rate of *An. gambiae *s.s, was significantly higher for treatment huts than for the control hut (P < 0.001). When comparing unwashed nets, a significantly higher mortality rate of *An. gambiae *s.s was recorded for unwashed PermaNet^® ^3.0 than for unwashed PermaNet^® ^2.0 (Additional file 1, Table S1) (P = 0.005). However, for washed nets, there was no statistical difference between the mortality rates *of An. gambiae *s.s for washed PermaNet^® ^2.0, washed PermaNet^® ^3.0 and the CTN (P = 0.337). For the other *Anopheles *species (*Anopheles nili, Anopheles pharoensis, Anopheles ziemanni*), the highest mortality rate was recorded with washed PermaNet^® ^2.0 (67.3%) and unwashed PermaNet^® ^3.0 (55.6%).

For *Mansonia *spp, the untreated control had the lowest mortality rate, followed by the washed PermaNet^® ^3.0 although this was significantly greater than the untreated control (P < 0.001); there was no significant difference between the mortality rates of the other treatment arms (P = 0.05). The highest mortality rate with *Mansonia *spp was recorded with unwashed PermaNet^® ^3.0 (69.0%) and PermaNet^® ^2.0 washed 20x (64.3%) (Additional file 3, Table S3). For pyrethroid-resistant *Culex *species, the highest mortality rate was observed with unwashed PermaNet^® ^3.0 (51.6%) (Additional file 2, Table S2).

For *Culex *spp, a significantly higher mortality rate was recorded with unwashed PermaNet^® ^3.0 (51.6%) compared to the other treatment arms (P < 0.001) with the lowest mortality recorded for the untreated net (6.6%) (P < 0.001). Meanwhile, a significantly higher mortality rate of *Culex *spp was recorded with unwashed PermaNet^® ^3.0 compared to the other treatment arms (P < 0.001). The mortality rate of unwashed PermaNet^® ^3.0 against *An. gambiae s.s *was significantly higher than the one of washed PermaNet^® ^3.0 (P = 0.020). The mortality rates of washed PermaNet^® ^2.0 and washed PermaNet^® ^3.0 were not statistically different from mortality rates with the CTN (P > 0.05).

Washing activities did not affect the efficacy of PermaNet^® ^2.0 against resistant *An. gambiae *s.s, resistant *Culex *spp or *Mansonia *spp as there was no significant difference between the mortality recorded before and after washing of this net (*An. gambiae *s.s, P = 0.558; *Culex *spp, P = 0.297, and *Mansonia *spp, P = 0.236). However, washing significantly reduced the efficacy of PermaNet^® ^3.0, as demonstrated by the statistically significant difference recorded between the mortality rates of 20x washed and unwashed PermaNet^® ^3.0 against pyrethroid-resistant *An. gambiae *s.s (P < 0.001), resistant *Culex *spp (P < 0.001) and *Mansonia *spp (P = 0.005).

### Blood feeding

The number of blood fed mosquitoes caught was very low because all nets tested were intact. The highest blood feeding rates for *An. gambiae *s.s were recorded with the CTN (8.6%), which was statistically similar to those recorded with unwashed PermaNet^® ^3.0 and unwashed PermaNet^® ^2.0 (P = 0.528), washed PermaNet^® ^3.0 and washed PermaNet^® ^2.0 (P = 0.336). The blood feeding rate of the untreated net for *An. gambiae *s.s was equal to 8.5%.

### Bioassays and knockdown rates

#### Mean KD and mortality rates against Kisumu strain

**Figure 1:**
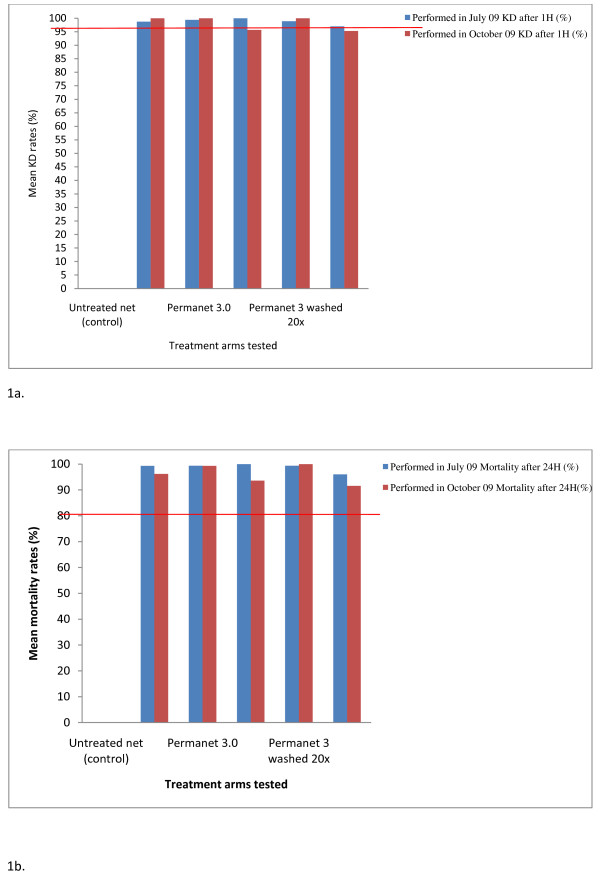
**(a & b): Mean Knock down (KD) and Mortality rates recorded after performing laboratory tests with each treatment arm against *An. gambiae *s.s. Kisumu strain at the beginning (July 09) and at the end of the trial (October 09)**.

Each treatment arm (unwashed PermaNet^®^2.0, washed PermaNet^®^2.0, unwashed PermaNet^® ^3.0, washed PermaNet^® ^3.0, washed and unwashed CTN, and untreated net) was bio-assayed before and after the trial. All treatments including the washed CTN showed a mean KD rate over the threshold of 95% and a mean mortality rate >80%, with the exception of the untreated net (Figure 1). The unwashed PermaNet^® ^3.0 gave a mean KD rate of 99.2% and 99.8% and a mean mortality rate of 99.1% and 99.5% from the sides and roof panels, respectively, whereas for washed PermaNet^® ^3.0 a mean KD rate of 99.1% and 99.5% and a mean mortality rate of 99.8% and 99.9% were recorded from the sides and roof panels, respectively.

#### Mean KD and mortality rates against pyrethroid-resistant wild caught *An. gambiae *s.s

**Figure 2:**
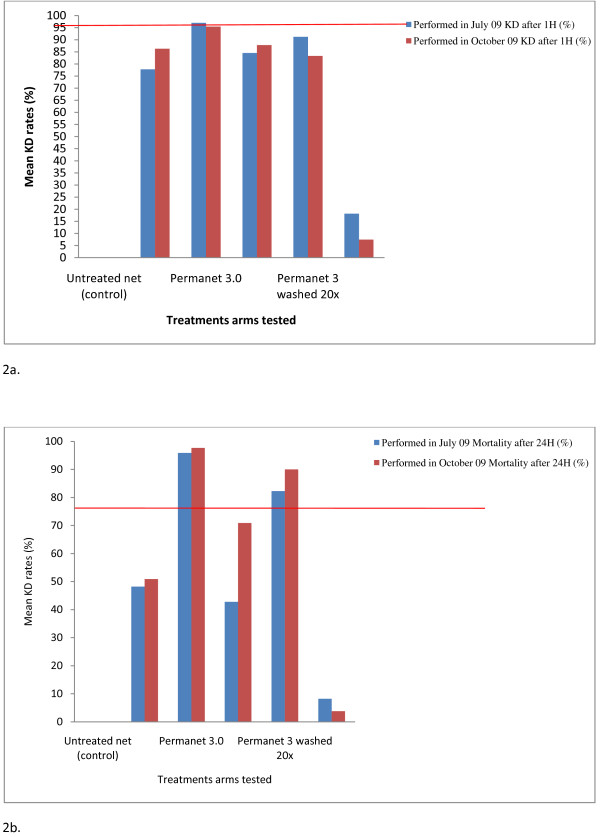
**(a & b): Mean Knock down (KD) and Mortality rates recorded after performing laboratory tests with each treatment arm against resistant *An. gambiae *wild strain at the beginning (July 09) and at the end of the trial (October 09)**.

The high levels of insecticide resistance in *An. gambiae *s.s mosquitoes collected in the study area was confirmed by the low mortality rates in WHO susceptibility tests with DDT, deltamethrin and permethrin (2.9%, 10.6% and 43.9% mortality, respectively). Cone bioassays performed with wild resistant *An. gambiae *s.s. mosquitoes in July and October 2009 showed a mean KD rate < 95% and a mean mortality rate < 80% for all treatment arms, with the exception of unwashed PermaNet^® ^3.0 (KD 95.8% and mortality 97.0%) (Figure 2).

For this unwashed PermaNet^® ^3.0, bioassays on side and roof panels yielded a mean KD of 94.3% and 98.6% and a mean mortality rate of 93.5% and 99.5%, respectively. For washed PermaNet^® ^3.0 a mean KD of 82.3% and 91.2% and a mean mortality rate of 83.3% and 90.0% were recorded for side and roof panels, respectively. The mean KD and mortality rate of pyrethroid resistant *An. gambiae s.s *recorded for the unwashed CTN were 95.7% and 81.6%, respectively.

## Discussion

The excito-repellency effect of all the treated nets was consistently high (≥ 50%) compared to the control. Mosquitoes were affected by pyrethroid on the nets, corroborating previous findings that pyrethroid-impregnated nets provide an irritant barrier against susceptible and resistant *An. gambiae *s.s mosquitoes, due to the excito-repellency and knock-down effects of pyrethroids [14]. The absence of holes in the nets, for ethical reasons, could have impacted the excito-repellency effect of all the treated nets, as sleepers would be accessible to *An. gambiae s.s*, *Culex sp, Mansonia *sp and other mosquitoes when under holed nets. Access to a blood meal may then reduce the excito-repellency rate recorded with each net. However, in areas with pyrethroid resistance, such as Benin [15], Burkina Faso and Cameroon [6], even when holes were present in the nets, the mortality rates recorded were low (30-40%) whereas in susceptible area such as Tanzania, mortality were above 80% [16] confirming the fact that mosquitoes resistance to pyrethroid reduced efficacy of nets. Low blood feeding rates were recorded for all treatment arms because nets were tested intact.

Cone bioassays against resistant *An. gambiae s.s *showed that the KD rate was > 95% and the mortality was > 80%, only with unwashed PermaNet^® ^3.0, although with washed 20x PermaNet^® ^3.0 a mortality rate > 80% was recorded against resistant *An. gambiae *s.s. Four out of five of the cone bioassays were performed on the polyester sides of washed PermaNet^® ^3.0 coated with high dose of deltamethrin (85 mg/m^2^). Previous studies conducted in sub-Saharan Africa have shown that the deltamethrin content remained high in the polyester sides of washed PermaNet^® ^3.0 even after 20 washes [6,17]. Chemical analysis of deltamethrin and PBO content on nets used in this study was not conducted.

The highest mortality rates against *An. gambiae s.s *(55.0%) and *Culex sp *(51.6%) were recorded with unwashed PermaNet^® ^3.0; mortality rates recorded with the other treatment arms were low (less than 40%) and did not differ significantly. The high dose of deltamethrin alone in the side panels of unwashed PermaNet^® ^3.0 could explain the significant difference in mortality of resistant *An. gambiae s.s *with this net compared to the other treatment arms. A previous study showed that the loss of deltamethrin after washing is very low in the side panels of PermaNet^® ^2.0 [6] where the dose of deltamethrin was initially equal to 55 mg/m^2 ^(±25%) before washing. That could partially explain why in these studies unwashed and washed PermaNet^® ^2.0 performed equally [17] confirming results reported in this study even if none measurement related to the deltamethrin and PBO doses was done before and after nets washing in the study.

The loss of efficacy of washed PermaNet^® ^in this study could be due to the resistance of *An. gambiae s.s *to pyrethroids, as in susceptible area such as Tanzania, the mortality rates recorded are usually above 80% even with washed PermaNet^® ^2.0 [16]. Consequently, washing is considered to be a more important loss mechanism for coated nets than for incorporated nets. Over the course of 20 washes, there was a weak partial loss of activity against the resistant *An. gambiae s.s *and *Culex spp *with PermaNet^® ^3.0 in the experimental hut trial, which was supported by bio-efficacy data indicating a reduction in mortality rates detected via cone bioassays on washed PermaNet 3.0. Probably the loss of deltamethrin after 20 washings in the polyester sides of PermaNet^® ^3.0, combined with the high pyrethroid resistance rates in *An. gambiae s.s *and *Culex spp *explains the low mortality rate recorded with washed PermaNet^® ^2.0 and PermaNet^® ^3.0. Results from cone bio-assays showed that the mortality rate recorded with 20x washed PermaNet 3.0 in the roof and sides and 20x washed PermaNet 2.0 against resistant *An. gambiae *was equal to 86% and 57%, respectively. Similar bioassays results were reported in Ladji, in southern Benin where *An. gambiae s.s *M form species were resistant to pyrethroids and DDT, with high frequency of *kdr *and metabolic resistance. In Benin, similar mortality rates were observed with *An. gambiae *tested in WHO susceptibility kits with deltamethrin (43.9% vs. 45.2%) [15]. The current study demonstrated that washed and unwashed PermaNet 2.0, washed PermaNet 3.0 and the conventionally-treated washed net (CTN) performed equally, while unwashed PermaNet 3.0 was associated with increased mortality. This is consistent with a recent study carried out in Benin, West Africa, which showed that although unwashed PermaNet^® ^3.0 was associated with a higher mortality than unwashed PermaNet 2.0 or unwashed Olyset, once washed, PermaNet 2.0 and 3.0 performed similarly [15]. In Vietnam where resistance did not seem to be affecting net efficacy, all PermaNet arms were performing slightly better than conventionally treated nets washed until just before exhaustion [17]. In Burkina Faso, in Vallee du Kou where the *kdr *mutation frequency was high (> 80%), results showed a strong reduction of LLIN efficacy. In this area, a significantly higher mortality and blood feeding inhibition was associated with unwashed and washed PermaNet^® ^3.0 compared to unwashed and washed PermaNet^® ^2.0 [18].

This study raises concerns that resistance appears to be having an impact on the efficacy of the nets in Ivory Coast (<40% mortality), in southern Benin (40 to < 30% mortality) with deltamethrin lambda-cyhalothrin [5,15] treated nets and in Burkina Faso (<40% mortality) [6] compared to >80% mortality in Tanzania where the vectors are susceptible [16]. Resistance mechanisms in *Anopheles gambiae *from the village of Ladji in Benin include *kdr *and metabolic mechanisms [5,19]. Resistance due to the involvement of P450s and *kdr *[15,20] has undermined attempts at malaria control with deltamethrin residual spraying in southern Africa caused by *An. funestus *[21]. Elevated P450 activity in a strain of *An. gambiae *from Cameroon has been found to reduce the efficacy of permethrin-treated netting in laboratory tests [22,23]. However, recent cone bio-assays performed on PermaNet 2.0 and PermaNet 3.0 after eight months use with metabolic resistant *An. gam*b*iae *and *Anopheles arabiensis *from northern Cameroon showed very high mortality rates (Koudou *et al*, unpublished data). Enzyme-based pyrethroid resistance mechanisms, such as elevated esterases and/or P450's, combined mechanisms such as *kdr *and metabolic resistance, and other mechanisms such as reduced penetration of insecticide, may be more of an obstacle to the control of malaria vectors in Cote d'Ivoire [24].

Washing of PermaNet^® ^2.0 and PermaNet^® ^3.0 did not affect their deterrent effects against resistant *An. gambiae s.s *or *Culex *sp. However, the deterrent effect, i.e. the reduction in the number of mosquitoes entering the hut, may not be a reliable indicator of ITN efficacy, as within the same geographical area this index has varied considerably with different vectors and nets (including PermaNet^® ^2.0) between experiments, e.g. from zero to 70.0% against *kdr*-based pyrethroid resistant *An. gambiae s.s *[25,26].

## Conclusion

To conclude, the present study showed that unwashed PermaNet^® ^3.0 caused higher mortality against resistant *An. gambiae s.s *and *Culex spp*. than washed and unwashed PermaNet^® ^2.0, washed PermaNet^® ^3.0 and the CTN. Village trials currently ongoing in Côte d'Ivoire and Cameroon in *kdr *and metabolic resistant areas, respectively, will allow the research team to conclude whether the enhanced efficacy of unwashed PermaNet^® ^3.0 over PermaNet^® ^2.0 and the CTN observed against both resistant *An. gambiae s.s *and *Culex spp *during the hut trial is enough to control highly pyrethroid-resistant malaria vector populations at the community level. The additive impact of unwashed PermaNet^® ^3.0 over PermaNet^® ^2.0 and CTN is confirmed by the significantly increased killing effect on both resistant *An. gambiae s.s *and *Culex spp *mosquitoes even if the mortality rate recorded with this net was not high (< 60%).

## Competing interests

The authors declare that they have no competing interests.

## Authors' contributions

BGK: designed experiments, coordinated field activities, collected and analysed data, wrote and revised the paper; AAK: designed the study and participated in the coordination of the field activities and revised the paper; DM: contributed to the study design and revised the paper; JH: participated in the design of the study and revised the paper. All authors have read and agreed with the contents of the submitted manuscript.

## Supplementary Material

Additional file 1 Summary of results obtained for *An. gambiae s.s*. (12 weeks) in experimental huts (Yaokoffikro, Côte d'Ivoire) Click here for file

Additional file 2 Summary of results obtained for *Culex sp *(12 weeks) in experimental huts (Yaokoffikro, Côte d'Ivoire) Click here for file

Additional file 3 Summary of results obtained for *Mansonia sp *(12 weeks) in experimental huts (Yaokoffikro, Côte d'Ivoire) Click here for file

## References

[B1] WHO World malaria report 2009 World Health Organization, Geneva http://www.who.int/malaria/world_malaria_report_2009/en/index.html 78 pp

[B2] Snow RW Guerra CA Noor AM Myint HY Hay SI The global distribution of clinical episodes of *Plasmodium falciparum *malaria Nature 2005 434 214 217 10.1038/nature03342 15759000 PMC3128492

[B3] Lopez AD Mathers CD Ezzati M Jamison DT Murray CJL Global and regional burden of disease and risk factors, 2001 systematic analysis of population health data Lancet 2006 367 1747 1757 10.1016/S0140-6736(06)68770-9 16731270

[B4] Lengeler C Insecticide-treated bed nets and curtains for preventing malaria Cochrane Database Syst Rev 2004 CD000363 10.1002/14651858.CD000363.pub2 15106149

[B5] N'Guessan R Corbel V Akogbeto M Rowland M Reduced efficacy of insecticide-treated nets and indoor residual spraying for malaria control in pyrethroid resistance area, Benin Emerg Infect Dis 2007 13 199 206 10.3201/eid1302.060631 17479880 PMC2725864

[B6] Corbel V Chabi J Dabiré Roch K Etang J Nwane P Pigeon O Akogbeto M Hougard JM Field efficacy of a new mosaic long-lasting mosquito net (PermaNet^® ^3.0) against pyrethroid resistant malaria vectors: a multi centre study in Western and Central Africa Malar J 2010 9 113 20423479 10.1186/1475-2875-9-113 PMC2877060

[B7] Koudou BG Adja AM Matthys B Doumbia M Cissé G Koné M Tanner M Utzinger J Pratiques agricoles et transmission du paludisme dans deux zones éco-épidemiologiques au centre de la Côte d'Ivoire Bull Soc Pathol Exot 2007 100 124 126 17727036

[B8] Koudou BG Doumbia M Janmohamed N Tschannen AB Tanner M Hemingway J Utzinger J Relationship between malaria transmission parameters and season in two agro-ecological settings of central Côte d'Ivoire Ann TropMed Parasitol 2010 104 109 121 10.1179/136485910X12607012374154 20406578

[B9] Chandre F Darriet F Manga L Akogbeto M Faye O Mouchet J Guillet P Status of pyrethroid resistance in *Anopheles gambiae *s.l Bull World Health Organ 1999 77 230 234 10212513 PMC2557627

[B10] Koudou GB Malone D Hemingway J Village scale testing of PermaNet® 2.0 and PermaNet® 3.0 to establish insecticide resistance breaking efficacy against An. gambiae s.s 2011 Interim report, ESAC meeting, Liverpool

[B11] Asidi AN Asidi AN N'Guessan R Koffi AA Curtis CF Hougard JM Chandre F Darriet F Zaim M Rowland MK Experimental hut evaluation of bed nets treated with an organophosphate (chlorpyrifosmethyl) or a pyrethroid (lambdacyalothrin) alone and in combination against insecticide-resistant *Anopheles gambiae *and *Culex quinquefasciatus *mosquitoes Malar J 2005 4 25 10.1186/1475-2875-4-25 15918909 PMC1156935

[B12] WHO Guidelines for laboratory and field testing of long-lasting insecticidal mosquito nets 2005 WHO/CDS/WHOPES/GCDPP/200511. World Health Organization, Geneva

[B13] Mattingly PF The mosquitoes of Ethiopian Region 1971 Suteliffe ed, London 184

[B14] Kolaczinski JH Fanello C Herve JP Conway DJ Carnevale P Curtis CF Experimental and molecular genetic analysis of the impact of pyrethroid and non-pyrethroid insecticide impregnated bednets for mosquito control in an area of pyrethroid resistance Bull Entomol Res 2000 90 125 132 10948372 10.1017/s0007485300000237

[B15] N'Guessan R Boko P Odjo A Chabi J Akogbeto M Rowland M Control of pyrethroid and DDT resistant *Anopheles gambiae *by application of indoor residual spraying or mosquito nets treated with a long-lasting organophosphate insecticide, chlorpyrifos-methyl Malar J 2010 9 44 10.1186/1475-2875-9-44 20141626 PMC2831012

[B16] Tungu P Magesa S Maxwell C Malima R Masue D Sudi W Myamba J Pigeon O Rowland M Evaluation of PermaNet 3.0 a deltamethrin-PBO combination net against *Anopheles gambiae *and pyrethroid resistant *Culex quinquefasciatus *mosquitoes: an experimental huts trial in Tanzania Malar J 2010 9 21 10.1186/1475-2875-9-21 20085631 PMC2817703

[B17] Van Bortel W Chinh VD Berkvens D Speybroeck N Trung HD Coosemans M Impact of insecticide-treated nets on wild pyrethroid resistant *Anopheles epiroticus *population from southern Vietnam tested in experimental huts Malar J 2009 8 248 10.1186/1475-2875-8-248 19874581 PMC2781025

[B18] Dabire RK Diabate A Baldet T Pare-Toe L Guiguemde RT Ouedraogo JB Skovmand O Personal protection of long lasting insecticide-treated nets in areas of *Anopheles gambiae *s.s. resistance to pyrethroids Malar J 2006 5 12 10.1186/1475-2875-5-12 16472385 PMC1402300

[B19] Corbel V Chandre F Brengues C Akogbeto M Lardeux F Hougard JM Guillet P Dosage-dependent effects of permethrin-treated nets on the behaviour of *Anopheles gambiae *and the selection of pyrethroid resistance Malar J 2004 3 22 10.1186/1475-2875-3-22 15242513 PMC471558

[B20] Donnelly MJ Corbel V Weetman D Wilding CS Williamson MS Black WC Does kdr genotype predict insecticide-resistance phenotype in mosquitoes? Trends Parasitol 2009 25 213 219 10.1016/j.pt.2009.02.007 19369117

[B21] Hargreaves K Koekemoer LL Brooke B Hunt RH Mthembu J Coetzee M *Anopheles funestus *resistant to pyrethroid insecticides in South Africa Med Vet Entomol 2000 14 181 189 10.1046/j.1365-2915.2000.00234.x 10872862

[B22] Etang J Chandre F Guillet P Manga L Reduced bio-efficacy of permethrin EC impregnated bed nets against an *Anopheles gambiae *strain with oxidase-based pyrethroid tolerance Malar J 2004 3 46 10.1186/1475-2875-3-46 15569394 PMC538265

[B23] Chouaibou M Simard F Chandre F Etang J Darriet F Hougard JM Efficacy of bifenthrin-impregnated bed nets against *Anopheles funestus *and pyrethroid-resistant *Anopheles gambiae *in North Cameroon Malar J 2006 5 77 10.1186/1475-2875-5-77 16961938 PMC1584243

[B24] Vulule JM Beach RF Atieli FK McAllister JC Brogdon WG Roberts JM Mwangi RW Hawley WA Elevated oxidase and esterase levels associated with permethrin tolerance in Anopheles gambiae from Kenyan villages using permethrin-impregnated nets Med Vet Entomol 1999 13 239 244 10.1046/j.1365-2915.1999.00177.x 10514048

[B25] Hougard JM Corbel V N'Guessan R Darriet F Chandre F Akogbéto M Baldet T Guillet P Carnevale P Traoré-Lamizana M Efficacy of mosquito nets treated with insecticide mixtures or mosaics against insecticide resistant *Anopheles gambiae *and *Culex quinquefasciatus *(Diptera: Culicidae) in Côte d'Ivoire Bull Entomol Res 2003 93 491 498 14704095 10.1079/ber2003261

[B26] Guillet P N'Guessan R Darriet F Traoré-Lamizana M Chandre F Carnevale P Combined pyrethroid and carbamate 'two-inone' treated mosquito nets: field efficacy against pyrethroid-resistant *Anopheles gambiae *and *Culex quinquefasciatus* Med Vet Entomol 2001 15 105 112 10.1046/j.1365-2915.2001.00288.x 11297094

