# Supplementary material for: Efficacy of PermaNet® 2.0 and PermaNet® 3.0 against insecticide-resistant Anopheles gambiae in experimental huts in Côte d'Ivoire
Source: Malar J. 2011 Jun 23;10:172. doi: 10.1186/1475-2875-10-172 (PMC3141592; doi:10.1186/1475-2875-10-172)
Supplement: Additional file 1 — Summary of results obtained for An. gambiae s.s. (12 weeks) in experimental huts (Yaokoffikro, Côte d'Ivoire) [file 1475-2875-10-172-S1.DOC]

**Additional file 1: Summary of results obtained for *An. gambiae s.s.* (12 weeks) in experimental huts (Yaokoffikro, Côte d’Ivoire)**

| Entomological indicators | Untreated  net | PermaNet® 3.0  unwashed | PermaNet® 2.0  unwashed | PermaNet® 3.0  washed 20 x | PermaNet® 2.0  washed 20 x | CTN |
| --- | --- | --- | --- | --- | --- | --- |
| Total females caught | 796 ^a^ | 303 ^b^ | 317 ^b^ | 313 ^b^ | 281 ^b^ | 279 ^b^ |
| females caught/night | 13.3 | 5.1 | 5.3 | 5.2 | 4.7 | 4.6 |
| Deterrence (%) | – | 62.1 | 60.4 | 60.1 | 64.4 | 64.6 |
|  |  |  |  |  |  |  |
| Total females veranda | 271 | 154 | 186 | 157 | 166 | 146 |
| Exophily (%) | 34.0^a^ | 50.8^b^ | 58.7^b^ | 50.2^b^ | 59.1^b^ | 52.3^b^ |
| 95% Confidence limits | 29.5 – 38.9 | 43.0 – 58.9 | 52.0 – 67.4 | 41.1 – 56.8 | 49.6 – 66.1 | 43.7 – 60.4 |
| Induced Exophily (%) | – | 66.8 | 74.3 | 65.0 | 72.8 | 67.8 |
|  |  |  |  |  |  |  |
| Total females dead | 54 | 166 | 110 | 100 | 107 | 110 |
| Overall mortality (%) | 6.8^a^ | 54.8^c^ | 34.7^b^ | 31.9^b^ | 38.1^b^ | 39.4^b^ |
| 95% Confidence limits | 4.2 – 9.5 | 46.5 – 63.2 | 26.3 – 42.2 | 24.5 – 40.0 | 29.8 – 47.2 | 30.8 – 48.3 |
| Corrected for control (%) | – | 57.7 | 38.9 | 36.2 | 42.1 | 43.3 |

^a^ Letters in the same row sharing a letter superscript do not differ significantly (P > 0.05)
