# Supplementary material for: Efficacy of PermaNet® 2.0 and PermaNet® 3.0 against insecticide-resistant Anopheles gambiae in experimental huts in Côte d'Ivoire
Source: Malar J. 2011 Jun 23;10:172. doi: 10.1186/1475-2875-10-172 (PMC3141592; doi:10.1186/1475-2875-10-172)
Supplement: Additional file 2 — Summary of results obtained for Culex sp (12 weeks) in experimental huts (Yaokoffikro, Côte d'Ivoire) [file 1475-2875-10-172-S2.DOC]

**Additional file 2: Summary of results obtained for *Culex sp* (12 weeks) in experimental huts (Yaokoffikro, Côte d’Ivoire)**

| Entomological indicators | Untreated  net | PermaNet® 3.0  unwashed | PermaNet® 2.0  unwashed | PermaNet® 3.0  20 washes | PermaNet® 2.0  20 washes | CTN |
| --- | --- | --- | --- | --- | --- | --- |
| Total females caught | 385 ^a^ | 173 ^b^ | 200 ^b^ | 188 ^b^ | 182 ^b^ | 185 ^b^ |
| females caught/night | 6.4 | 2.9 | 3.3 | 3.1 | 3.0 | 3.1 |
| Deterrence (%) | – | 55.1 | 47.5 | 51.1 | 53.0 | 52.1 |
|  |  |  |  |  |  |  |
| Total females veranda | 134 | 78 | 106 | 106 | 85 | 89 |
| Exophily (%) | 34.8^a^ | 45.1^b^ | 53.0^b^ | 56.4^b^ | 46.7^b^ | 48.1^b^ |
| 95% Confidence limits | 27.9 – 36.7 | 36.6 – 54.8 | 44.8 – 62.4 | 47.1 – 64.1 | 38.5 – 57.1 | 39.6 – 57.3 |
| Induced Exophily (%) |  | 62.4 | 69.7 | 71.7 | 64.4 | 65.0 |
|  |  |  |  |  |  |  |
| Total females dead | 26 | 90 | 71 | 45 | 79 | 63 |
| Overall mortality (%) | 6.6^a^ | 51.6^c^ | 33.4^b^ | 23.4^b^ | 44.6^b^ | 33.7^b^ |
| 95% Confidence limits | 4.3 – 9.8 | 43.3 – 59.9 | 26.3 – 41.3 | 17.3 – 30.9 | 36.3 – 53.1 | 26.5 – 41.7 |
| Corrected for control (%) | – | 54.6 | 37.5 | 28.1 | 48.1 | 37.8 |

^a^ Letters in the same row sharing a letter superscript do not differ significantly (P > 0.05)
