# Supplementary material for: Efficacy of PermaNet® 2.0 and PermaNet® 3.0 against insecticide-resistant Anopheles gambiae in experimental huts in Côte d'Ivoire
Source: Malar J. 2011 Jun 23;10:172. doi: 10.1186/1475-2875-10-172 (PMC3141592; doi:10.1186/1475-2875-10-172)
Supplement: Additional file 3 — Summary of results obtained for Mansonia sp (12 weeks) in experimental huts (Yaokoffikro, Côte d'Ivoire) [file 1475-2875-10-172-S3.DOC]

**Additional file 3: Summary of results obtained for *Mansonia sp*** (12 weeks) in experimental huts (Yaokoffikro, Côte d’Ivoire)

| **Entomological indicators** | **Untreated**  **net** | **PermaNet® 3.0**  **unwashed** | **PermaNet® 2.0**  **unwashed** | **PermaNet® 3.0**  **20 washes** | **PermaNet® 2.0**  **20 washes** | **CTN** |
| --- | --- | --- | --- | --- | --- | --- |
| Total females caught | 486 ^a^ | 566 ^a^ | 605 ^a^ | 604 ^a^ | 600 ^a^ | 575 ^a^ |
| females caught/night | 40.5 | 47.2 | 50.4 | 50.3 | 50.0 | 47.9 |
| Deterrence (%) | – | 116.8 | 123.5 | 125.0 | 127.2 | 119.7 |
|  |  |  |  |  |  |  |
| Total females veranda | 173 | 268 | 264 | 264 | 280 | 268 |
| Exophily (%) | 35.6^a^ | 47.3^b^ | 43.6^b^ | 43.7^b^ | 46.7^b^ | 46.6^b^ |
| 95% Confidence limits | 31.4 – 40.2 | 43.4 – 51.7 | 39.2 – 47.4 | 39.6 – 47.6 | 42.4 – 50.9 | 42.6 – 50.8 |
| Induced Exophily (%) |  | 62.0 | 57.8 | 58.1 | 61.2 | 61.2 |
|  |  |  |  |  |  |  |
| Total females dead | 76 | 387 | 384 | 315 | 388 | 347 |
| Overall mortality (%) | 14.9^a^ | 69.0^b^ | 64.1^b^ | 51.8^c^ | 64.3^b^ | 61.2^b^ |
| 95% Confidence limits | 10.9 – 20.0 | 63.4 – 74.1 | 58.3 – 69.4 | 46.1 – 57.4 | 58.4 – 69.8 | 55.4 – 66.7 |
| Corrected for control (%) | – | 73.0 | 68.8 | 58.1 | 68.9 | 66.2 |

^a^ Letters in the same row sharing a letter superscript do not differ significantly (P > 0.05
